# Diversity formation, education, and training in medical education

**Published:** 2018-11-12

**Authors:** Manish Ranpara

**Affiliations:** 1 Deparatment of Family Medicine, University of Calgary, Alberta, Canada

The Declaration of Geneva, widely regarded as the modern Hippocratic Oath and the guiding light for physicians was written in 1948 in response to the atrocities of World War II. The Declaration builds on pillars of ethical medicine and highlights the fiduciary duties of physicians to their patients free from any bias and discrimination. Seventy years since its creation, I am writing this letter perplexed that the medical profession remains unfaithful to some of its principles. In this letter, I will focus on lack of physician education in diversity and cultural awareness that may be contributing to this deficiency.

Barinder Singh and colleagues^1^ report that Canadian medical residents have limited training in cross-cultural care. They found that family medicine and psychiatry residents reported more core-cultural training and assessment than did their peers in other specialties. Alarmingly, residents across specialties felt relatively skilled in this area despite the lack of training. This may mean one of several things: the residents felt that the skill is intrinsic; the residents learnt this skill from another source; or the residents were unaware of their limitations in this area. It is likely the latter. It is imperative that universities, training programs, and senior staff ensure that cultural competence is taught, learned, and assessed, especially since insensitivity and discrimination are linked to poorer health outcomes in patients.

Similarly, Brenda Beagan and colleagues^2^ conducted interviews of family physicians to explore perceptions of care of Lesbian, Gay, Bisexual, Transgender, or Queer (LGBTQ) women. Their thematic analysis revealed that physicians varied in their approach to incorporating gender identity into their care. They concluded that better education, including an explanation on the difference between generalizations and stereotyping, was needed to address the healthcare needs of the LGBTQ community more effectively.

So, how can students and residents cultivate their knowledge and skills in diversity? As medical schools and programs around the world become aware of the importance of diversity training, several methods have been used to teach and develop these skills. Some of these methods include classroom instruction, online modules, standardized patients, peer-assessment, multi-source feedback, and reflective exercises. While it is commendable that medical educators are trying to address this knowledge gap through changes in medical curricula, more rigorous assessment is needed. Perhaps there should be cross-cultural communication OSCEs at the end of medical school, and more scenarios relating to diversity in licensing examinations. This would help students realize the importance of these topics (after all, how important can it be if it is not rigorously assessed?) and focus more of their time and effort on learning these skills.

Medical education has changed over the years, and it continues to do so. We must effectively teach and thoroughly assess our foundational principles of ethics and equality to serve the needs of our ever more diverse patient population better.
